# Identification of the Germline Mutation Profile in Esophageal Squamous Cell Carcinoma by Whole Exome Sequencing

**DOI:** 10.3389/fgene.2019.00047

**Published:** 2019-02-18

**Authors:** Jiaying Deng, Xiaoling Weng, Junyi Ye, Daizhan Zhou, Yun Liu, Kuaile Zhao

**Affiliations:** ^1^Department of Radiation Oncology, Fudan University Shanghai Cancer Center, Shanghai, China; ^2^Department of Oncology, Shanghai Medical College, Fudan University, Shanghai, China; ^3^Shanghai Fudan University Shanghai Cancer Center and Institutes of Biomedical Sciences, Collaborative Innovation Center of Cancer Medicine, Shanghai Medical College, Fudan University, Shanghai, China; ^4^Burning Rock Biotech, Guangzhou, China; ^5^Key Laboratory of Arrhythmias of the Ministry of Education of China, East Hospital, Tongji University School of Medicine, Shanghai, China; ^6^Institute of Medical Genetics, Tongji University, Shanghai, China; ^7^Institutes of Biomedical Sciences, Fudan University, Shanghai, China

**Keywords:** whole exome sequencing, ESCC, germline mutation, VUS, clinical features

## Abstract

**Background:** Esophageal squamous cell carcinoma (ESCC) is associated with poor prognosis and occurs with high frequency in China. The germline mutation profile in ESCC remains unclear, and therefore, the discovery of oncogenic alterations in ESCC is urgently needed. This study investigates the germline mutation profile and reveals associations among genotype-environment interactions in ESCC.

**Methods:** Whole exome sequencing and follow-up analysis were performed in 77 matched tumor-normal ESCC specimens to examine the germline profiles. Additionally, associations among genotype-environment interactions were investigated.

**Results:** We identified 84 pathogenic/likely pathogenic mutations and 51 rare variants of uncertain significance (VUS). Twenty VUS with InterVar evidence of a score of moderate pathogenicity (PM) 2/PM2+ supporting pathogenicity (PP) 1 were found to have pathogenic significance. CYP21A2 was the most frequently mutated gene, and the p.Gln319^*^ variant was identified in 6.5% (5/77) of patients. The TP53 p.V197E mutation, located within the DNA binding domain, was found in 1.3% (1/77) of patients. In total, the 11.7% (9/77) of individuals with homologous recombination (HR) VUS were more likely to have well-differentiated tumors than those without (*P* = 0.003). The degree of lymph node metastasis was correlated with homologous recombination deficiency (HRD) and VUS group (*P* < 0.05). Moreover, the 10.4% (8/77) of individuals with mismatch repair (MMR) VUS had a higher tumor mutational burden (TMB), although the correlation was not significant.

**Conclusions:** Our study identified the germline mutation profiles in ESCC, providing novel insights into the molecular pathogenesis of this disease. Our results may also serve as a useful resource for the exploration of the underlying mechanism of ESCC and may provide information for the prevention, diagnosis and risk management of ESCC.

## Introduction

Esophageal cancer (EC) has a poor prognosis and is the sixth leading cause of cancer-related death and the eighth most common cancer worldwide (Domper Arnal et al., 2015). Esophageal squamous cell carcinoma (ESCC) is the major histological type of esophageal cancer, and more than half of all esophageal cancer cases worldwide occur in China (Chen et al., 2016). Despite the tremendous efforts toward and progression of surgical techniques and other treatment approaches in ESCC, recurrence after esophagectomy or definitive chemoradiotherapy is still high (Enzinger and Mayer, 2003). Currently, understanding the molecular mechanisms that underlie tumourigenesis is necessary to identify additional diagnostic markers that contribute to ESCC. The discovery of drugs that target specific oncogenic alterations for the treatment of ESCC is urgent.

Genomic studies have investigated the landscape of ESCC based on whole genome sequencing (WGS) and whole exome sequencing (WES) (Lin et al., 2014; Song et al., 2014). Genomic alterations, especially somatic mutations, were more distinct based on the sequencing data. In our previous study, we compared genomic alterations between Asian and Caucasian patient populations (Deng et al., 2017). We identified ESCC-associated genes (TP53, NOTCH1, and PIK3CA) widely reported in previous studies. In Asian patients, EP300 and NFE2l2 were found to have higher mutation frequencies, while CSMD3 was associated with better prognosis. The findings revealed the molecular basis underlying the striking racial disparities in ESCC. However, to our knowledge, the germline susceptibility gene mutation profile in ESCC remains unclear. In this study, we performed WES on 77 ESCC specimens and adjacent normal tissues (ANT) to identify the germline mutation signature in Chinese ESCC subjects. This study not only reports the germline profiles in ESCC but also reveals associations among genotype-environment interactions.

## Materials and Methods

### Sample Acquisition

Human primary ESCC tissues and corresponding adjacent non-tumor tissues (located 5 cm from the tumors) were collected from patients diagnosed with ESCC who underwent surgery as a primary treatment at Fudan University Cancer Center (Shanghai, China) between September 2007 and June 2011. Tumor tissues were snap frozen in liquid nitrogen immediately after surgical resection and stored at −80°C until further analysis. The clinicopathological features of the patients, including age, sex, smoking history, alcohol use history, tumor site, family history, differentiation, and tumor/node/metastasis (TNM) stage, were collected from inpatient medical records. The pathological features were evaluated independently by pathologists according to the TNM staging system of the American Joint Committee on Cancer (AJCC 7th edition). The study protocol was approved by the Ethics Committee of Fudan University Cancer Center. Samples were collected from a tissue bank, which obtained written informed consent from the participants.

### Whole Exome Sequencing

Genomic DNA was extracted from tissue specimens using a QIAamp DNA kit (Qiagen), and libraries were then prepared using protocols recommended by Illumina. Briefly, 1 μg of DNA was sheared into short fragments (200~300 bp) using a Covaris S220 ultrasonicator. The DNA fragments were then end repaired to generate adenylated 3′ ends. Adaptors with barcode sequences were then ligated to both ends of the fragments, and E-Gels were used to select DNA fragments of the targeted size. Next, 10 PCR cycles were performed, and the resulting product was purified. Whole exome capture was performed using a TruSeq Exome Enrichment kit (Illumina) according to the manufacturer's protocol with slight modifications. After the Illumina sequencing libraries were amplified with 10 PCR cycles, capture probes were added, and the reaction mixtures were incubated at 65°C for 24 h. The hybridized mixtures were then amplified with an additional 10 PCR cycles, and the validated DNA libraries were sequenced on an Illumina sequencing system (Illumina HiSeq 2500).

### Data Processing and Mutation Calling

Read pairs (FASTQ data) generated from the sequencing system were first trimmed and filtered using Trimmomatic (Bolger et al., 2014). The resulting reads were aligned to the hg19 reference genome using Burrows-Wheeler Aligner (BWA) (Li and Durbin, 2009). Variants were called using a variant calling pipeline based on SAMtools mpileup output (Li et al., 2009). Germline mutations were called in both normal and cancer samples. Variant annotation was performed using ANNOVAR (Wang et al., 2010). SIFT was applied to annotate and predict the functions of the mutation variants (Ng and Henikoff, 2001). Genes with q values (false discovery rate, FDR) of ≤ 0.05 were considered significantly mutated. We considered non-synonymous SNPs for the calculation of the tumor mutational burden (TMB), which was defined as the number of mutations/Mb of sequenced DNA.

### Clinical Pathological and Genetic Data

The homologous recombination (HR) repair pathway and the DNA mismatch repair (MMR) pathway are common signaling pathways explored in germline mutation analysis. The list of analyzed genes, including common and important genes related to germline mutations in various cancers, was shared by the AstraZeneca medical department. The HR and MMR pathways play vital roles in the development of a variety of cancers. We integrated all variants of uncertain significance (VUS) from the 77 ESCC cases and determined alterations in the homologous recombination arm of the DNA repair pathway (GO_RECOMBINATIONAL_REPAIR) and the DNA mismatch repair pathway (GO_MISMATCH_REPAIR).

**Figure 1:**
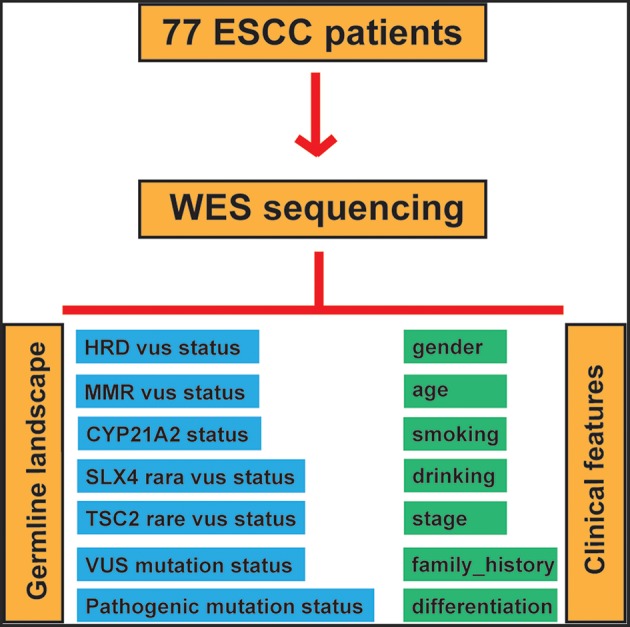
Study workflow.

To assess the correlation between clinicopathological features and germline alterations, we conducted univariate analysis, and correlations were determined using the Fisher, Wilcoxon and Pearson tests according to the different variable types. Then, significantly different factors were included in a multivariate analysis to strengthen the findings. Germline alterations in the patients included VUS in HR pathway genes, VUS in MMR pathway genes, rare VUS in SLX4, rare VUS in TSC2, pathogenic mutations in CYP21A2, the rare VUS carrier group and the pathogenic mutation carrier group. The workflow of our study is illustrated in Figure 1.

## Results

### Population Characteristics

The clinical characteristics of the patients are summarized in Table 1 [updated from our previous study (Deng et al., 2017)]. In total, 65 male patients and 12 female patients were included in our study. Of these patients, 46 (59.7%, 46/77) had a history of smoking, 36 (46.8%, 36/77) had a history of alcohol abuse, and 12 (15.6%, 12/77) had a family history of ESCC. The median follow-up time was 35.5 months. All 77 patients were diagnosed with stage T2 disease, 4 had well-differentiated tumors, 45 had moderately differentiated tumors, and 28 presented with poorly differentiated tumors. Sixty-two patients were classified as TNM stage II, and 15 were classified as TNM stage III.

**Table 1:** Clinicopathological parameters of the study participants.

	**N**	**Percent (%)**
**SEX**		
Male	65	84.4
Female	12	15.6
**AGE**		
≤50	11	14.3
51–60	34	44.2
>60	32	41.6
**SMOKING**		
Yes	46	59.7
No	31	40.3
**ALCOHOL USE**		
Yes	36	46.8
No	41	53.2
**FAMILY HISTORY**		
Yes	12	15.6
No	65	84.4
**T STAGE**		
T2	77	100
**N STAGE**		
N1	64	82.1
N2	12	16.7
N3	1	1.3
**DIFFERENTIATION**		
Well	4	5.2
Moderate	45	58.4
Poor	28	36.4
**SITE (7th)**		
Upper thorax	17	22.1
Middle thorax	48	62.3
Lower thorax	12	15.6
**TNM STAGE**		
II	62	80.5
III	15	19.5

*This table is updated from our previous study (Deng et al., 2017)*.

### Germline Mutational Landscape of ESCC

**Figure 2:**
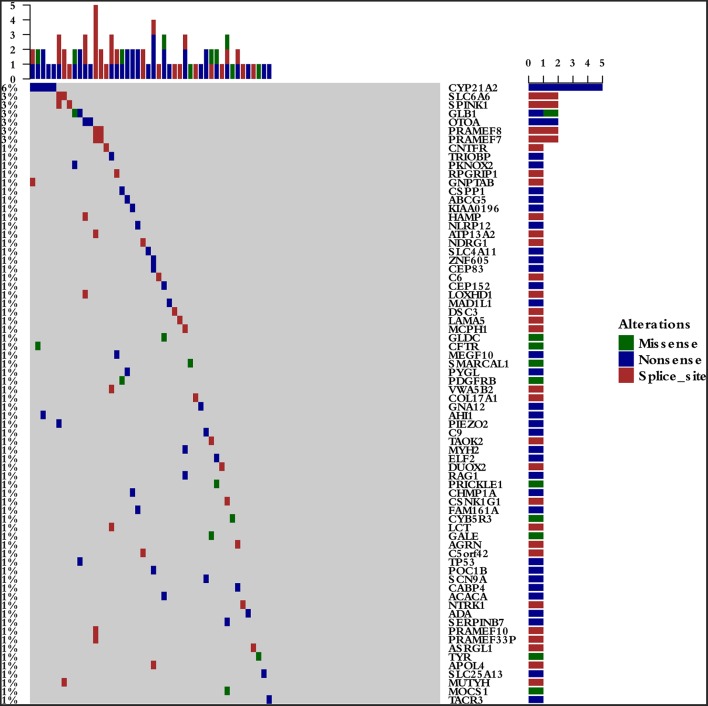
The 46 pathogenic/likely pathogenic germline mutations in the study cohort. The detection rates were 6.5% (5/77) for CYP21A2 p.Gln319^*^, 2.6% (2/77) for SPINK1 c.194+2T>C, 2.6% (2/77) for OTOA p.Glu1048^*^, 2.6% (2/77) for PRAMEF7/8 c.863+1G>T, and 2.6% (2/7) for PRAMEF10/33P c.866+2T>C.

**Figure 3:**
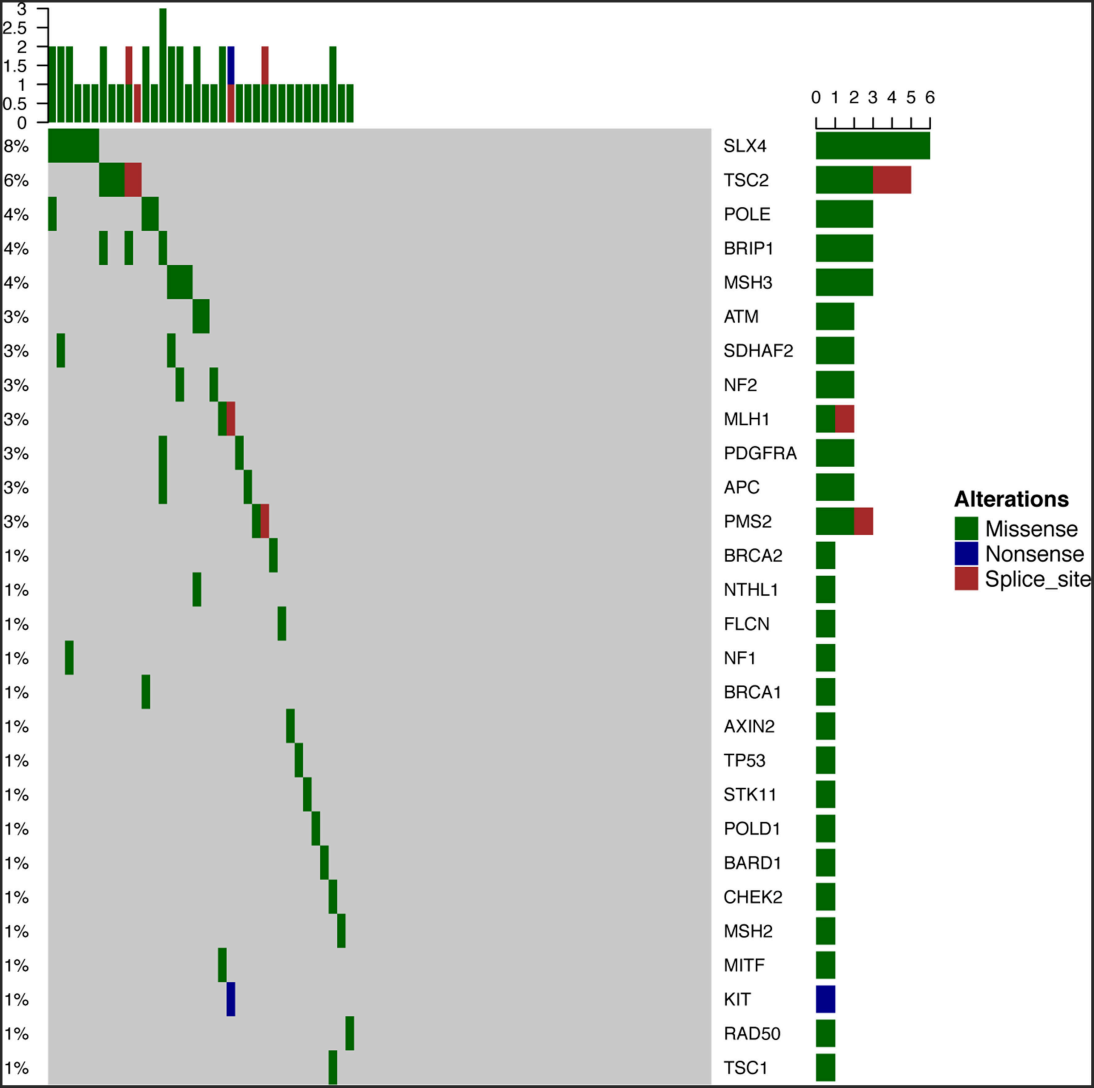
Fifty-one rare VUS among all samples. Fifty-one rare VUS with a frequency of ≤0.01, including SLX4, TSC2, MSH3, ATM, APC, and TP53, were identified.

To survey the mutations in susceptibility genes in esophageal squamous cell carcinoma, we sequenced whole exomes from all 77 individuals in the study cohort. The mean coverage in the WES data was 95%. We identified 84 pathogenic/likely pathogenic mutations in 46 individuals [(59.7%, 46/77)—a detection rate of 6.5% (5/77) for CYP21A2 p.Gln319^*^, 2.6% (2/77) for SPINK1 c.194+2T>C, 2.6% (2/77) for OTOA p.Glu1048^*^, 2.6% (2/77) for PRAMEF7/8 c.863+1G>T, and 2.6% (2/7) for PRAMEF10/33P c.866+2T>C] (Figure 2). Sanger sequencing was used to validate the results, with 100% concordance (Supplementary Figure 1). The detailed characteristics of the 84 pathogenic/likely pathogenic mutations carried in 46 individuals and the enriched pathways are summarized in Supplementary Table 1. Pathway analyses were performed using the Kyoto Encyclopedia of Genes and Genomes database (KEGG). The galactose metabolism, MAPK signaling pathway and central carbon metabolism in cancer pathways were enriched (Supplementary Table 1). Pathogenic mutations in CYP21A2 were the most prevalent, while the second most common mutations were in the SPINK1, OTOA, PRAMEF7/8, and PRAMEF10/33P genes. Table 2 summarizes the most frequently identified pathogenic mutations in CYP21A2, SPINK1, OTOA, PRAMEF7/8, and PRAMEF10/33P, which include 6 splice donor variants and 7 stop-gain variants.We also identified 67,468 VUS, of which 51 were rare VUS with a frequency of ≤ 0.01 (Figure 3). Rare VUS in SLX4 were the most prevalent, accounting for 7.8% (6/77) of missense mutation carriers. TSC2 was the second most commonly mutated gene, with a 6.5% (5/77) carrier rate including 3 missense mutations and 2 splice site variants. Two patients in the study cohort were carriers of the MSH3 p.A57_A58delins mutation supporting pathogenicity (PP), which was correlated with hereditary susceptibility to ESCC. Table 3 shows the 20 VUS with InterVar evidence of a score of moderate pathogenicity (PM) 2/PM2+PP1, which were likely to have pathogenic significance. Among these mutations, the TP53 missense mutation p.V197E, which is located in the DNA binding domain, was deleterious and demonstrated a probable contribution to ESCC progression (Table 4).

**Table 2:** Pathogenic/likely pathogenic germline variants in susceptibility genes.

**ID**	**Gene**	**Mutation_Type**	**HGVS_P**	**CHR**	**POS**	**REF**	**ALT**	**Intervar_evide**	**Pop_Freq**	**Degrees**
E07060	CYP21A2	Stop_gained	p.Gln319^*^	6	32008198	C	T	PVS1;PM2;PP3;	1.00E-04	Pathogenic
E11261	CYP21A2	Stop_gained	p.Gln319^*^	6	32008198	C	T	PVS1;PM2;PP3;	1.00E-04	Pathogenic
E12173	CYP21A2	Stop_gained	p.Gln319^*^	6	32008198	C	T	PVS1;PM2;PP3;	1.00E-04	Pathogenic
E12182	CYP21A2	Stop_gained	p.Gln319^*^	6	32008198	C	T	PVS1;PM2;PP3;	1.00E-04	Pathogenic
E12222	CYP21A2	Stop_gained	p.Gln319^*^	6	32008198	C	T	PVS1;PM2;PP3;	0.0001	Pathogenic
E09135	SPINK1	Splice_donor_variant	NA	5	147207583	A	G	PVS1;PP3;PP5	0.0035	Pathogenic
E11348	SPINK1	Splice_donor_variant	NA	5	147207583	A	G	PVS1;PP3;PP5	0.0035	Pathogenic
E08113	OTOA	Stop_gained	p.Glu1048^*^	16	21764434	G	T	PVS1;PM2;PP3	0	Pathogenic
E09207	OTOA	Stop_gained	p.Glu1048^*^	16	21764434	G	T	PVS1;PM2;PP3	0	Pathogenic
E09151	PRAMEF7	Splice_donor_variant	NA	1	13609242	C	A	PVS1;PM2	0	Likely pathogenic
E11172	PRAMEF7	Splice_donor_variant	NA	1	13609242	C	A	PVS1;PM2	0	Likely pathogenic
E09151	PRAMEF10	Splice_donor_variant	NA	1	13412814	T	C	PVS1;PM2	0	Likely pathogenic
E09151	PRAMEF10	splice_donor_variant	NA	1	13412814	T	C	PVS1;PM2	0	Likely pathogenic

*PVS, very strong pathogenicity; PM, moderate pathogenicity; PP, supporting pathogenicity. (The interpretation and grade of the sequence variants refer to PMID 25741868)*.

**Table 3:** Twenty VUS with susceptibility to be likely pathogenic.

**ID**	**Gene**	**Mutation type**	**HGVS_P**	**CHR**	**POS**	**REF**	**ALT**	**intervar_evide**	**Pop_Freq**	**Degrees**
E10335	TP53	Missense variant	p.Val197Glu	17	7578259	A	T	PM1;PM2;PP3	0	VUS
E09012	BRIP1	Missense variant	p.Gln102Arg	17	59934493	T	C	PM1;PM2;BP4	0	VUS
E09012	PDGFRA	Missense variant	p.Thr234Ile	4	55131158	C	T	PM1;PM2;PP3	0	VUS
E09012	APC	Missense variant	p.Leu662Ile	5	112173275	C	A	PM1;PM2;PP3	9.00E-04	VUS
E09117	POLE	Missense variant	p.Arg793His	12	133241978	C	T	PM1;PM2;PP3	1.22E-04	VUS
E09151	MSH2	Missense variant	p.Ile735Val	2	47703703	A	G	PM1;PM2;PP3	0.001	VUS
E09162	BRIP1	Missense variant	p.Leu340Phe	17	59878736	G	A	PM1;PM2;PP3	2.00E-04	VUS
E10164	PMS2	Missense variant	p.Pro771Gln	7	6017352	G	T	PM1;PM2;PP3	5.82E-05	VUS
E10168	NTHL1	Missense variant	p.Pro125Thr	16	2096134	G	T	PM1;PM2;PP3	5.80E-05	VUS
E10220	NF2	Missense variant	p.Ile487Leu	22	30074197	A	C	PM1;PM2;BP4	0	VUS
E10308	PMS2	Missense variant	p.Val302Phe	7	6031688	C	A	PM1;PM2;PP3	1.00E-04	VUS
E10315	CHEK2	Missense variant	p.Ser252Asn	22	29107934	C	T	PM1;PM2;PP3	0.001	VUS
E10354	NF2	Missense variant	p.Arg376Gln	22	30069262	G	A	PM1;PM2;PP3	1.14E-05	VUS
E11214	STK11	Missense variant	p.Arg42Leu	19	1207037	G	T	PM1;PM2;BP4	8.00E-04	VUS
E11235	BARD1	Missense variant	p.Arg99Lys	2	215657089	C	T	PM1;PM2;PP3	0	VUS
E11303	BRCA2	Missense variant	p.Ala2911Glu	13	32950906	C	A	PM2;PM3;PP5	0	VUS
E11348	TSC2	Missense variant	p.Val67Phe	16	2100461	G	T	PM1;PM2;PP3	0	VUS
E12047	MLH1	Missense variant	p.Ser368Leu	3	37067192	C	T	PM1;PM2;PP4	0.001	VUS
E12173	POLD1	Missense variant	p.Gly504Arg	19	50910363	G	C	PM1;PM2;PP5	0	VUS
E12269	MLH1	Missense variant	p.Val152Met	3	37050305	C	A	PM1;PM2;PP6	8.13E-06	VUS

VUS, variants of uncertain significance; PM, moderate pathogenicity; PP, supporting pathogenicity; BP, benign supporting pathogenicity. (The interpretation and grade of the sequence variants refer to PMID 25741868.)

**Table 4:** Characteristics of the identified rare VUS TP53 p.V197E.

**cDNA description**	**Protein description**	**Exon number**	**Effect**	**SIFT class**
c.590T>A	p.Val197Glu	exon 6	missense_variant	deleterious

*VUS, variants of uncertain significance*.

### Correlation of Clinical Features and Germline Mutations

**Figure 4:**
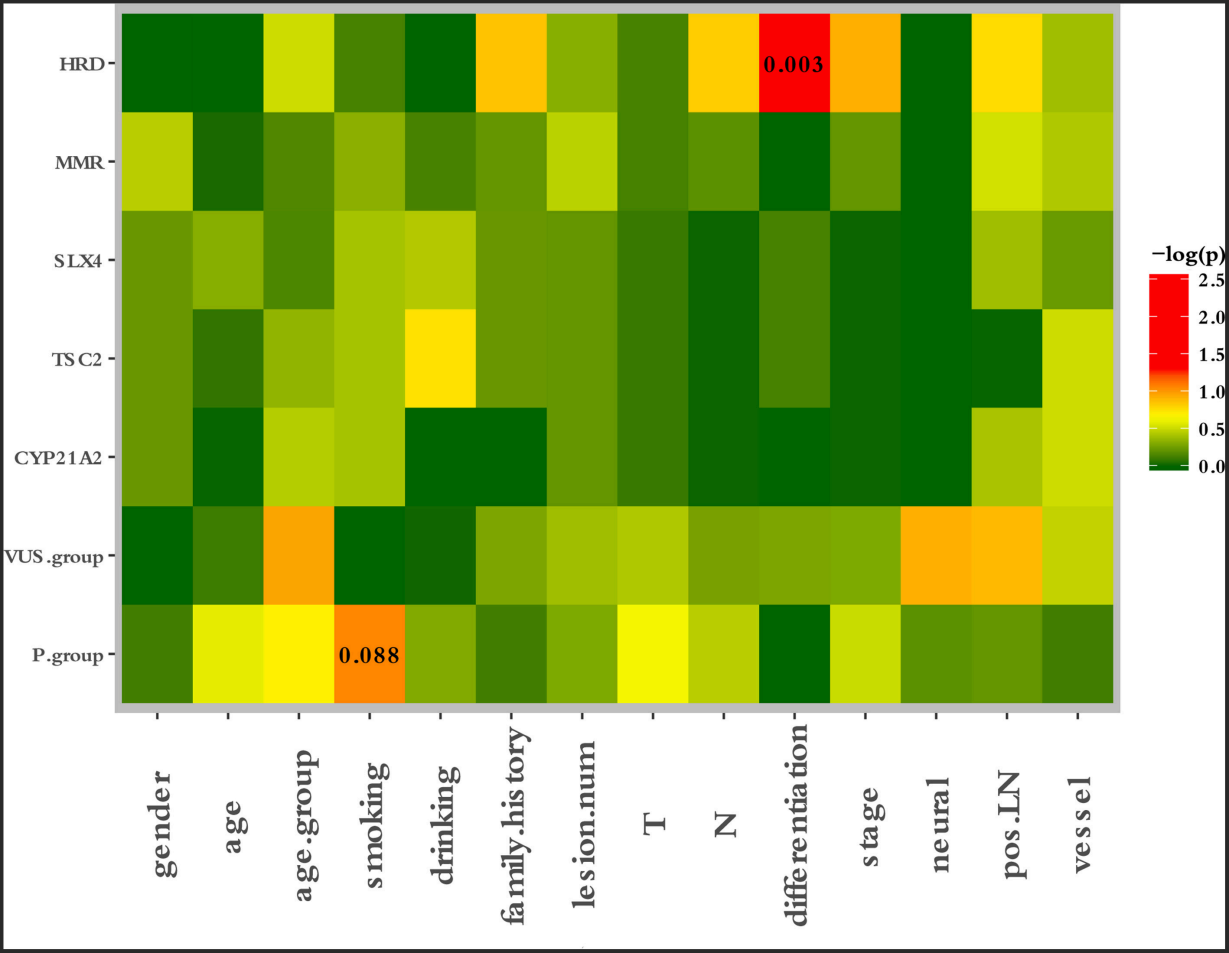
Correlation between clinical risk factors and germline mutation status. The patient characteristics of gender, age, age group, smoking history, drinking history, family history of ESCC, lesion number, T stage, N stage, differentiation, TNM stage, perineural invasion, LN positivity, and growth of new vessels were compared with the germline mutation status, which comprised the HRD VUS status, MMR VUS status, SLX4 rare VUS status, TSC2 rare VUS status, CYP21A2 pathogenic mutation status, VUS group, and P group.

**Figure 5:**
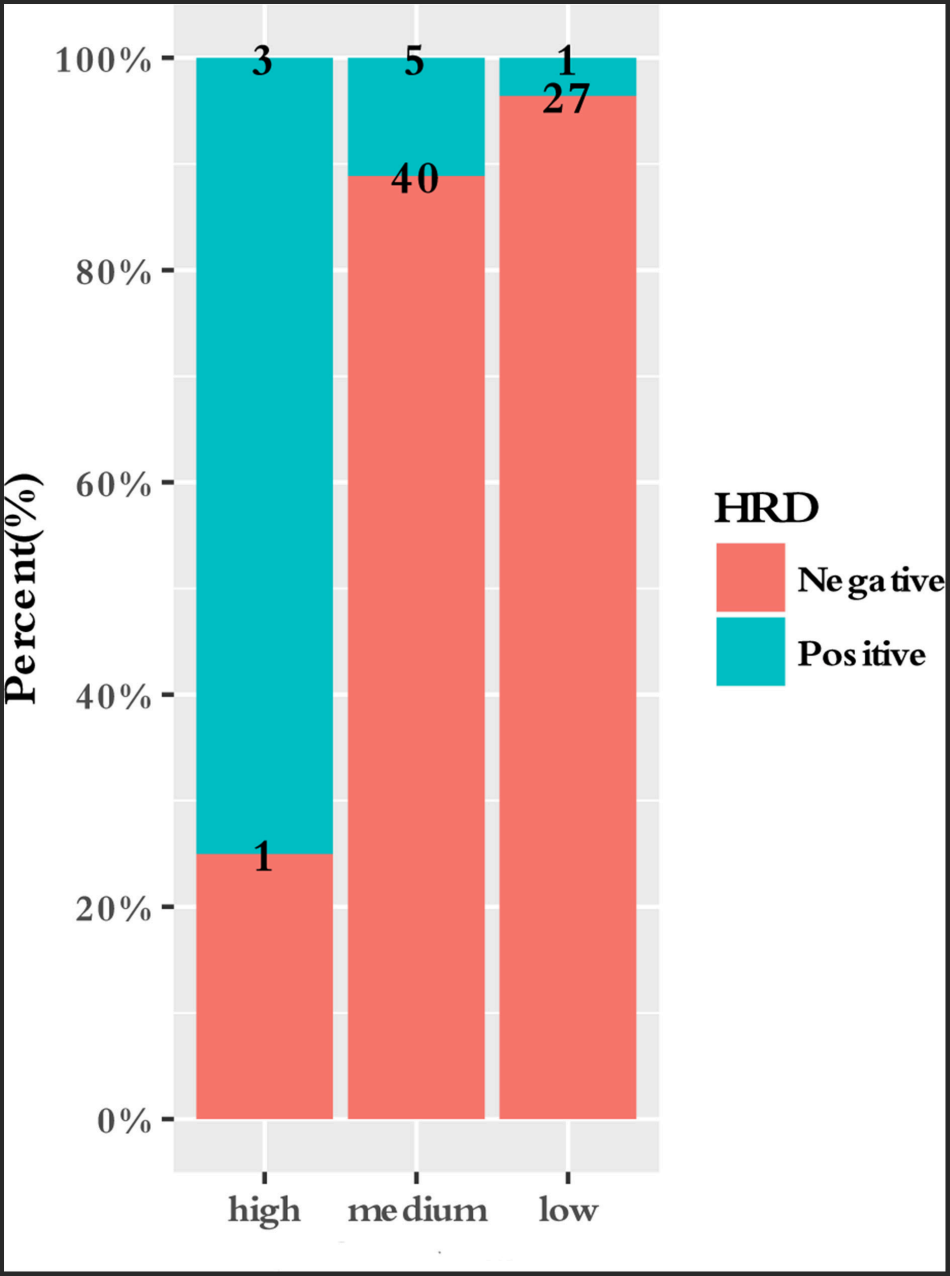
Correlation between differentiation and HRD VUS status. Patients who carried HRD VUS were more likely to have highly differentiated tumors than patients who did not (*P* = 0.003).

**Figure 6:**
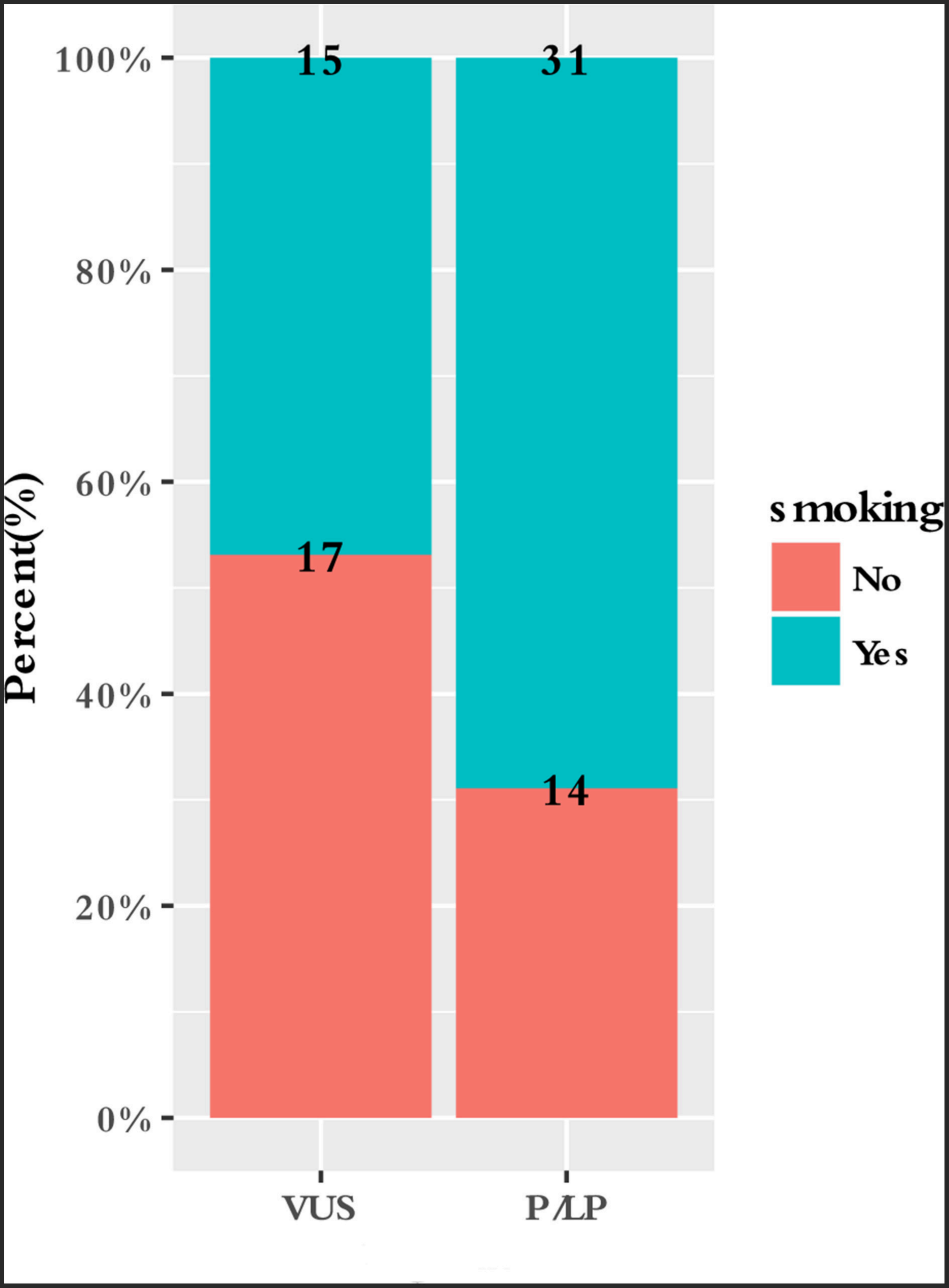
Correlation between smoking and germline mutation status.

To characterize the clinical features upon cancer diagnosis in the germline mutation carriers, we compared the clinical characteristics with the different mutation statuses. Among the 77 patients with ESCC, the patient characteristics of gender, age, age group, smoking history, drinking history, family history of ESCC, lesion number, T stage, N stage, differentiation, TNM stage, perineural invasion, LN positivity, and growth of new vessels were compared with the germline mutation status, which comprised homologous recombination deficiency (HRD) VUS status, MMR VUS status, SLX4 rare VUS status, TSC2 rare VUS status, CYP21A2 pathogenic mutation status, VUS_group, and P_group (Figure 4). In total, 11.7% (9/77) of the individuals were carriers of HRD VUS, and those patients were more likely to have well-differentiated tumors than moderately or poorly differentiated tumors (*P* = 0.003, Table 5 and Figure 5). A correlation existed between pathogenic/likely pathogenic mutations and cigarette consumption (Figure 6). Other cancer characteristics did not show statistically significant associations with germline mutation status. Moreover, the 10.4% (8/77) of individuals who were carriers of MMR VUS had a higher tumor mutational burden (TMB) than those who were not (Supplementary Table 2 and Supplementary Figure 2), although the non-significance of the difference may be due to the limited number of samples. Among those patients, the two who were carriers of MSH3 p.A57_A58delinsPP had a much higher TMB (5.82 and 4.47), and one patient who was a carrier of PMS2 p.S587N and p.V302F had a TMB of 6.47. Multivariate analyses showed that the degree of lymph node metastasis was correlated with HRD and VUS group (*P* = 0.03 and 0.04, respectively). A greater degree of lymph node metastasis occurred in patients with HRD and in those with VUS.

**Table 5:** Detailed information of 9 HRD VUS carriers.

**ID**	**Gene**	**Mutation type**	**Description**	**Age**	**Smoking**	**Alcohol**	**Family history**	**Differentiation**	**TNM stage**	**Degree**
E09012	BRIP1	Missense variant	p.Q102R	64	Yes	Yes	Yes	Yes	IIB	VUS
E09162	BRIP1	Missense variant	p.L340F	53	No	Yes	No	No	IIB	VUS
E10168	ATM	Missense variant	p.Q912E	70	Yes	No	No	No	IIB	VUS
E10199	BRIP1	Missense variant	p.D563G	48	Yes	Yes	Yes	Yes	IIB	VUS
E10315	CHEK2	Missense variant	p.S252N	64	Yes	Yes	No	No	IIB	VUS
E11221	ATM	Missense variant	p.E953K	65	No	No	No	No	IIB	VUS
E11235	BARD1	Missense variant	p.R99K	49	Yes	No	No	No	IIB	VUS
E11303	BRCA2	Missense variant	p.A2911E	65	No	No	No	No	IIB	VUS
E12115	BRCA1	Missense variant	p.A1199V	57	Yes	No	Yes	Yes	IIB	VUS

*HRD, homologous recombination deficiency; VUS, variant of uncertain significance*.

## Discussion

The current study describes an in-depth exome analysis of the germline mutational landscape of ESCC. We identified 84 pathogenic/likely pathogenic mutations and 51 rare VUS in our study cohort. We also found 20 VUS with InterVar evidence of a score of PM2/PM2+PP1, which were likely to have pathogenic significance. Our study provided germline mutation profiles in ESCC, which may serve as a useful resource for the exploration of the mechanisms underlying ESCC.

TP53 is a tumor suppressor that plays a key role in the cellular stress response (Vogelstein et al., 2000). TP53 has been detected in cancer biopsies by virtue of its high protein expression level, which is considered indicative of mutation (Petitjean et al., 2007; Vijayakumaran et al., 2015). In our study, the TP53 p.V197E mutation is located within the DNA binding domain and may contribute to ESCC progression, an observation that accounts for the improper DNA binding and disruption of transcriptional activity that was observed. The TP53 V197E mutant was also found to bind to PBF and promote colorectal tumorigenesis (Read et al., 2016).

In the current analysis, a correlation between cigarette use and germline mutations existed. Individuals carrying germline mutations who smoke cigarettes may be a high-risk population for esophageal cancer (Supplementary Figure 3). Cigarette smoking has been identified as an important pathogenic factor for ESCC (Domper Arnal et al., 2015). Polycyclic aromatic hydrocarbons and aromatic amines are major classes of carcinogens present in tobacco (Bartsch et al., 2000). These molecules are converted into DNA-reactive metabolites by cytochrome P450 (CYP)-related enzymes (Wu et al., 2002). CYP21A2 encodes a member of the cytochrome P450 enzyme superfamily. CYP21A2 p.Gln319^*^ was the most prevalent mutation in our study. Steroid 21-hydroxylase deficiency due to CYP21A2 gene mutations, including p.Gln319^*^, is seen in more than 90% of all congenital adrenal hyperplasia cases (Prado et al., 2017). We hypothesized that CYP21A2 p.Gln319^*^ may affect the metabolism of tobacco and participate in the development of ESCC (Supplementary Figure 3). It is interesting that the inhibition of CYP21A2 by 1 μM abiraterone was observed in a recent study (Malikova et al., 2017). Song et al found 3 SNPs in CYP19A1, which also encodes a cytochrome P450 enzyme, that were associated with a FH of UGI cancer in ESCC cases (*P* < 10^−5^) in a meta-analysis performed by the National Cancer Institute (NCI) and in the Henan GWAS data (Song et al., 2017). As described above, crosstalk between CYP21A2 p.Gln319^*^ and smoking may promote ESCC progression. The function of CYP21A2 in esophageal cancer requires further investigation, including *in vitro* and *in vivo* experiments.

We found that individuals who carried HRD VUS were more likely to have highly differentiated tumors than those who did not. Defects in the homologous recombination (HR) DNA repair pathway sensitize tumors to therapeutics that target this pathway. In breast cancer, patients with mutations in the HR pathway are more likely to achieve a pathologic complete response (pCR) than non-deficient patients (Telli et al., 2018). The literature shows that as many as one in four ovarian cancer cases exhibit germline mutations, the majority of which result in HRD (Frey and Pothuri, 2017). Our results were consistent with research in breast and ovarian cancer. The current study is the first report of HRD VUS and differentiation status in ESCC. Additional in-depth studies with larger numbers of cases are needed to investigate HRD VUS in ESCC.A high TMB is an emerging biomarker of sensitivity to immune checkpoint inhibitors and has been shown to be significantly associated with a response to PD-1 and PD-L1 blockade immunotherapy (Le et al., 2015; Rizvi et al., 2015). Chalmers ZR et al. found that recurrent promoter mutations in PMS2 are highly associated with increased TMB across a diverse cohort of 100,000 cancer cases with over 100 tumor types (Chalmers et al., 2017).

In our study, we found that individuals who carried MMR VUS have higher TMB than those who did not. E09211 and E12230, who carried MSH3 p.A57_A58delinsPP, had TMB of 5.82 and 4.47, respectively. E10308, who carried PMS2 p.S587N and p.V302F, had a TMB of 6.47. DNA mismatch repair (MMR) has been widely documented to play a vital role in the development of a variety of cancers. Studies have shown that both the loss of expression and overexpression of mismatch repair genes, including MLH1, MSH2, and PMS2, can be deleterious to genomic stability (Thibodeau et al., 1996; Qin et al., 1999; Gibson et al., 2006). Our results were consistent with those of previous studies, which demonstrated that loss-of-function mutations in mismatch repair pathway genes correlate with a high TMB (Duval and Hamelin, 2002; Peltomäki, 2003).

In conclusion, our results identified the profile of germline mutations that predispose individuals to ESCC, providing novel insights into the molecular pathogenesis of ESCC. These results may potentially play key roles in the prevention, diagnosis, and risk management of ESCC. A large sample size with a long follow-up is required in the near future to validate our observations.

## Author Contributions

JD: conceptualization, writing manuscript, and editing. XW: data curation, writing original draft and editing. JY: formal analysis, software and methodology. DZ and YL: sample sequencing and funding acquisition. KZ: funding acquisition, project administration and manuscript review.

### Conflict of Interest Statement

The authors declare that the research was conducted in the absence of any commercial or financial relationships that could be construed as a potential conflict of interest.
