# Supplementary material for: Identification of the Germline Mutation Profile in Esophageal Squamous Cell Carcinoma by Whole Exome Sequencing
Source: Front Genet. 2019 Feb 18;10:47. doi: 10.3389/fgene.2019.00047 (PMC6387948; doi:10.3389/fgene.2019.00047)
Supplement: Supplementary file 3 [file Data_Sheet_1.docx]

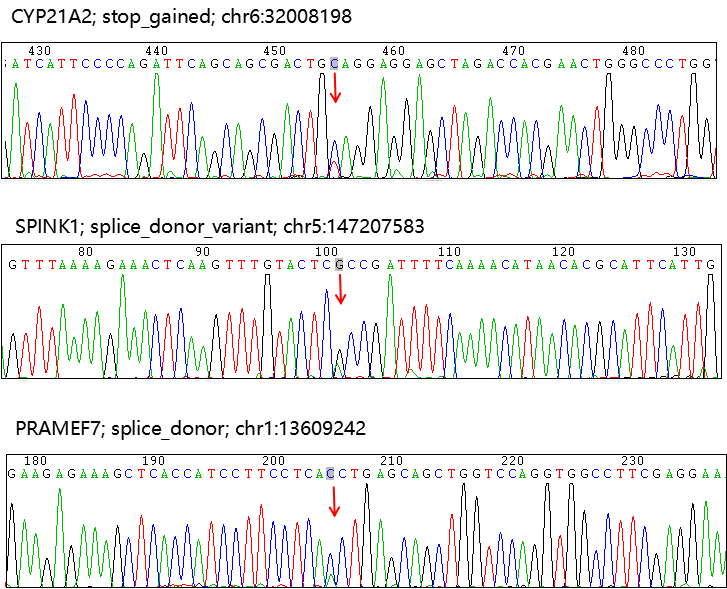


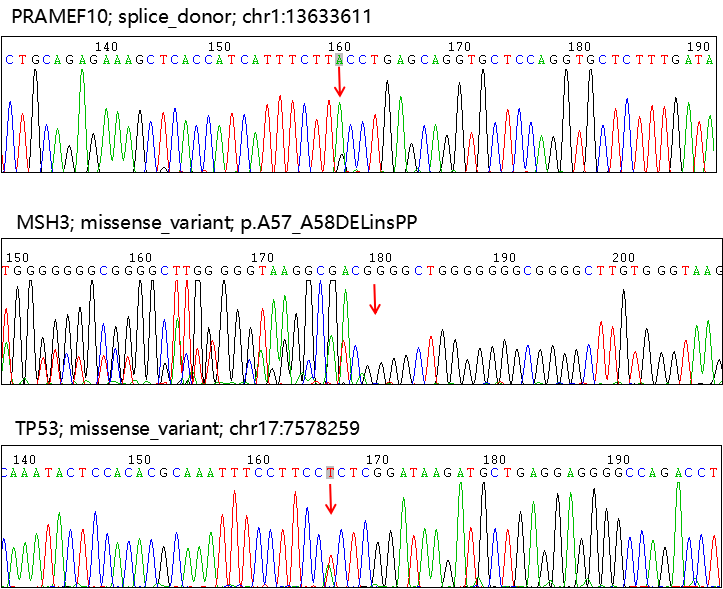


**Supplementary Figure 1.** Validation sequencing analysis of mutations in the current study.


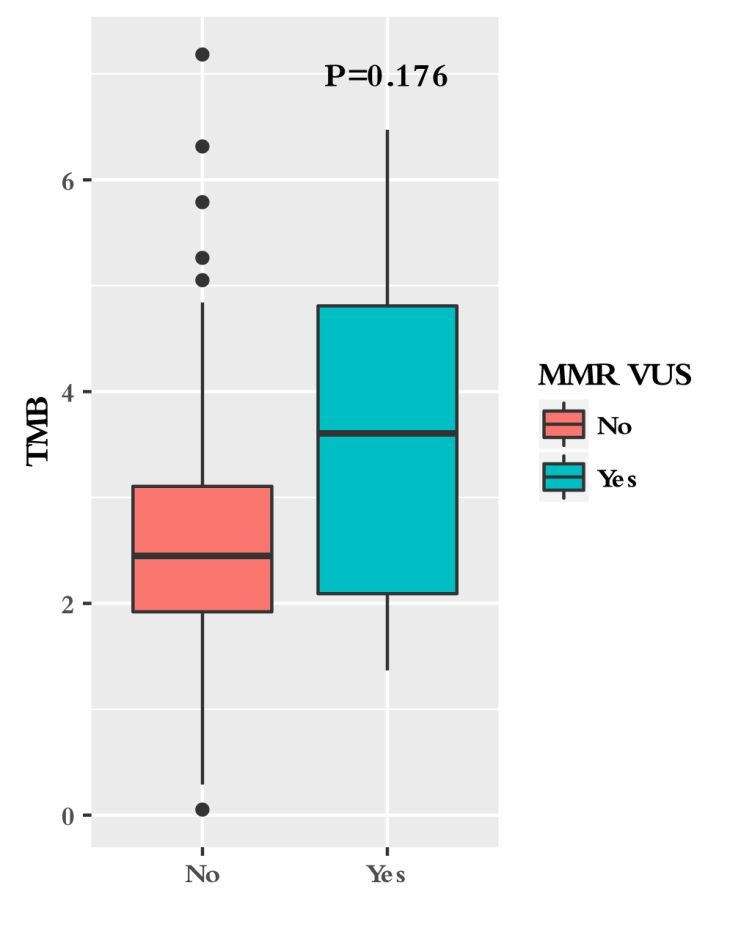


**Supplementary Figure 2.** Correlation between MMR VUS status and TMB.


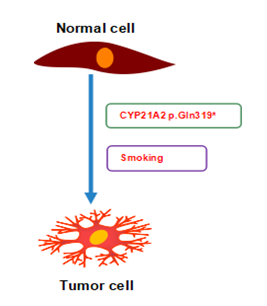


**Supplementary Fig. 3.** The crosstalk betweenCYP21A2p.Gln319* and smoking may promote ESCC progression.

CYP21A2 belongs to the cytochrome P450 superfamily of enzymes and CYP21A2p.Gln319* was identified in 6.5% (5/77) patients in our study. Cytochrome P450 (CYP)-related enzymes play roles in converting tobacco component polycyclic aromatic hydrocarbons and aromatic amines into DNA-reactive metabolites. The crosstalk betweenCYP21A2p.Gln319* and smoking may promote ESCC progression (Supplementary Fig. 3) and the function of CYP21A2 in esophageal cancer needs further investigation.
